# Relationships of Adiponectin with Markers of Systemic Inflammation and Insulin Resistance in Infants Undergoing Open Cardiac Surgery

**DOI:** 10.1155/2013/187940

**Published:** 2013-06-10

**Authors:** Yukun Cao, Ting Yang, Shiqiang Yu, Guocheng Sun, Chunhu Gu, Dinghua Yi

**Affiliations:** ^1^Department of Cardiovascular Surgery, Xijing Hospital, Fourth Military Medical University, No. 15, Changle West Road, Xi'an, Shaanxi 710032, China; ^2^Department of Oral Anatomy and Physiology and TMD, School of Stomatology, Fourth Military Medical University, Xi'an, Shaanxi 710032, China

## Abstract

*Background*. Insulin resistance and systemic inflammation frequently occur in infants undergoing cardiac surgery with cardiopulmonary bypass, while adiponectin has been demonstrated to have insulin-sensitizing and anti-inflammatory properties in obesity and type 2 diabetes mellitus. In this prospective study, we aimed to investigate the association of adiponectin with insulin resistance and inflammatory mediators in infants undergoing cardiac surgery with cardiopulmonary bypass. *Methods and Results*. From sixty infants undergoing open cardiac surgery, blood samples were taken before anesthesia, at the initiation of cardiopulmonary bypass and at 0, 6, 12, 24, and 48 hours after the termination of cardiopulmonary bypass. Plasma interleukin-6 (IL-6), tumor necrosis factor-alpha (TNF-**α**), and adiponectin levels were assessed in blood samples. Insulin resistance was measured by assessment of the insulin requirement to maintain euglycaemia and repeated measurements of an insulin glycaemic index. Insulin glycaemic index, IL-6, and TNF-**α** increased up to 3–8-fold 6 h after the operation. Adiponectin is negatively correlated with markers of systemic inflammation 6 h after CPB. *Conclusions*. Although the level of serum adiponectin decreased significantly, there was a significant inverse association of adiponectin with markers of systemic inflammation and insulin resistance in infants undergoing open cardiac surgery.

## 1. Introduction

Insulin resistance and systemic inflammation frequently occur in infants undergoing cardiac surgery with cardiopulmonary bypass (CPB). Insulin resistance presenting with increased blood glucose level (hyperglycemia) and decreased sensitivity to insulin increases morbidity and mortality in critically ill patients [1, 2]. Intensive insulin therapy aiming at euglycemia improves their clinical outcome [3–5]. In a recently published study involving patients undergoing cardiac surgery, intraoperative insulin resistance was associated with an increased risk of short-term adverse outcomes [6]. The inflammatory reaction and injury may contribute to the development of postoperative complications [7, 8]. The magnitude and duration of the systemic inflammatory response determine the development of tissue damage, multiorgan failure, or even death [9, 10]. Our previous studies have demonstrated that ameliorating insulin resistance attenuates the systemic inflammatory response in infants undergoing CPB [11]. 

 Adiponectin, a hormone derived from the adipose tissue, has been demonstrated to have insulin-sensitizing and anti-inflammatory properties in obesity and type 2 diabetes mellitus [12]. Recently adiponectin has also been shown to have a reverse correlation with insulin resistance and inflammatory mediators [13]. Studies on the relationship of adiponectin with insulin resistance and inflammatory mediators in infants undergoing cardiac surgery with cardiopulmonary bypass are scarce. The present study was undertaken to investigate the association of adiponectin with the development of insulin resistance and kinetic changes of inflammatory mediators in infants undergoing CPB.

## 2. Materials and Methods 

The present study has been approved by the Ethics Committee of Xijing Hospital, The Fourth Military Medical University, and performed according to the World Medical Association Declaration of Helsinki.

### 2.1. Patients

Patient population: infants aged between 6 months and 3 years undergoing open cardiac surgery with CPB for congenital heart disease were enrolled for the study at our hospital from June 2010 to August 2011. Detailed information was given to the parents preoperatively and their written consent was obtained. None of the infants had a history of diabetes mellitus. Exclusive criteria included preoperative liver and kidney disease or dysfunction, preoperative coagulation disorder, palliative or second operation, and impaired blood glucose levels. 

### 2.2. Measurements of Insulin Resistance

 Overnight fasting was advised for all patients on the preoperative day. Insulin resistance was recorded by the individual insulin requirements to maintain euglycemia. Blood glucose was monitored on an hourly basis and insulin infusion rate was adjusted to maintain glucose levels between 4.4 and 8.3 mmol/L. The infusion of insulin is a standard of care and started when the glucose concentration became higher than 8.3 mmol/L. An insulin glycaemic index (insulin × glucose/22.5) was calculated at each time point. 

### 2.3. Determination of Insulin, Adiponectin, IL-6, and TNF-*α* Levels

 Blood samples were taken at 7 time points for each patient as follows: before anesthesia (*T*1), at the initiation of CPB (*T*2), at the termination of CPB (*T*3), 6 h after CPB (*T*4), 12 h after CPB (*T*5), 24 h after CPB (*T*6), and 48 h after CPB (*T*7). Serum level of adiponectin was determined with a commercial enzyme-linked immunosorbent assay (R&D, Wiesbaden, Germany). Serum insulin levels were measured with an insulin kit (R&D Systems, Abingdon, UK). Plasma IL-6 and TNF-*α* levels were determined using commercially available ELISA kits (R&D Systems, Abingdon, UK) [14]. All enzyme-linked immunosorbent assay (ELISA) protocols were carried out according to kit guidelines. 

### 2.4. Statistical Analysis

**Figure 1:**
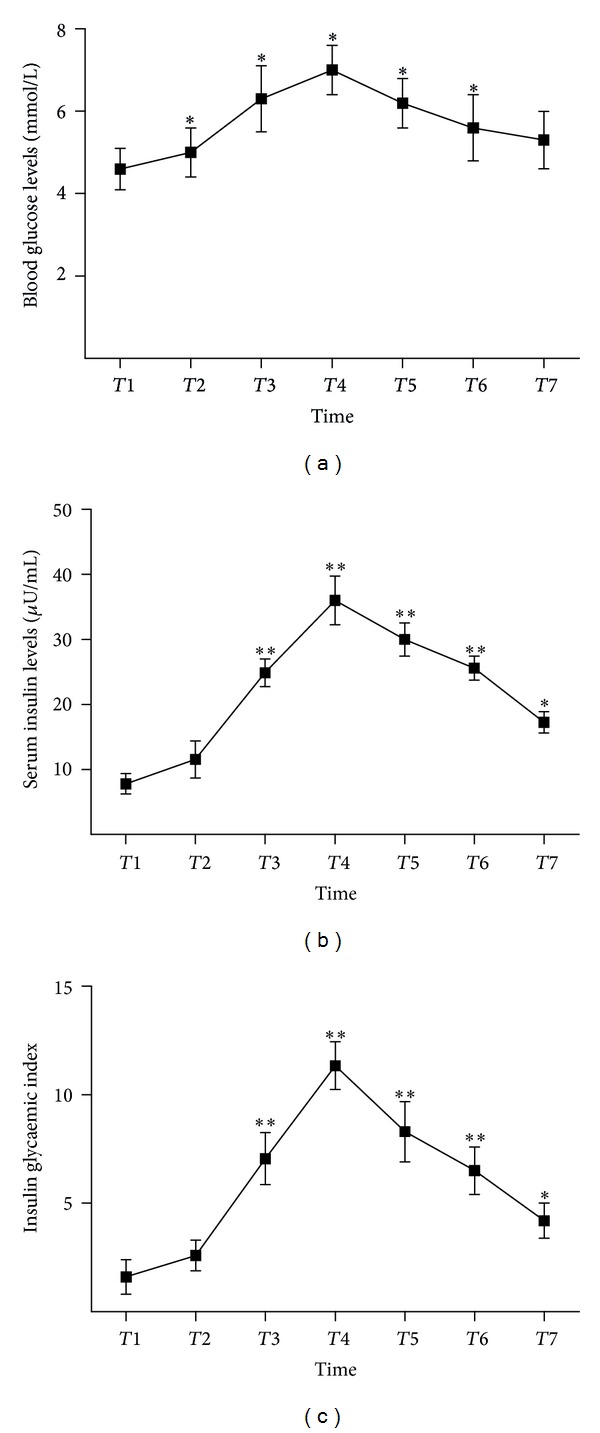
Changes in blood glucose levels, insulin levels, and insulin glycaemic index in the perioperative period. Reported significances (*P < 0.05, **P < 0.01 were calculated using pairwise comparisons with the preoperative level within a repeated measurement analysis of variance model for the respective parameter at different time points). The error bars designate the standard deviation (CPB, cardiopulmonary bypass; *T*: time; *T*1: before anesthesia; *T*2: initiation of CPB; *T*3: termination of CPB; *T*4: 6 h after CPB; *T*5: 12 h after CPB; *T*6: 24 h after CPB; *T*7: 48 h after CPB).

**Figure 2:**
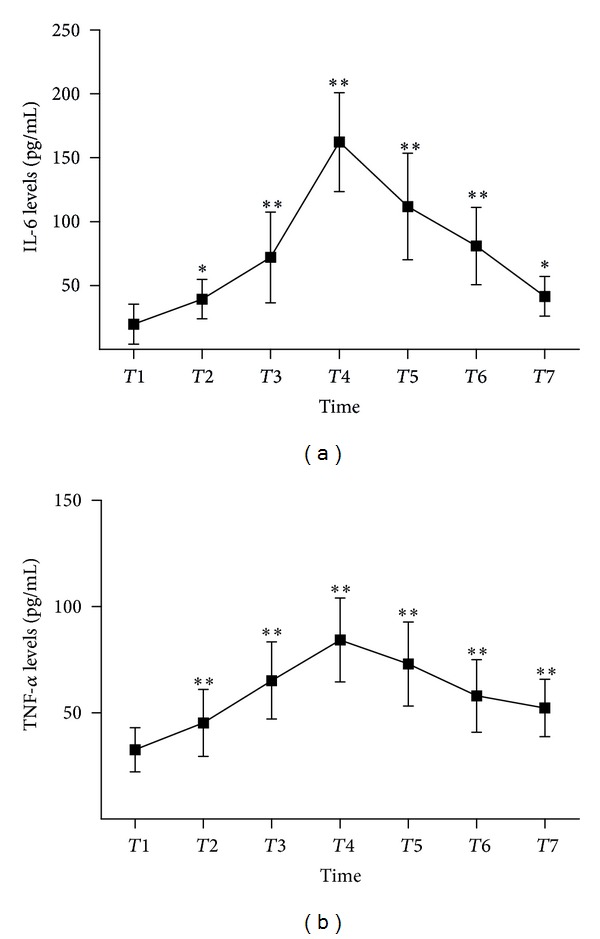
Pre- and postoperative TNF-*α* and IL-6 (*P < 0.05, **P < 0.01 compared with basal levels). The error bars designate standard deviation. IL-6 and TNF-*α* levels are higher than basal levels and did not normalize within the study period ((a) and (b)). (CPB: cardiopulmonary bypass; *T*: time; *T*1: before anesthesia; *T*2: initiation of CPB; *T*3: termination of CPB; *T*4: 6 h after CPB; *T*5: 12 h after CPB; *T*6: 24 h after CPB; *T*7: 48 h after CPB).

**Figure 3:**
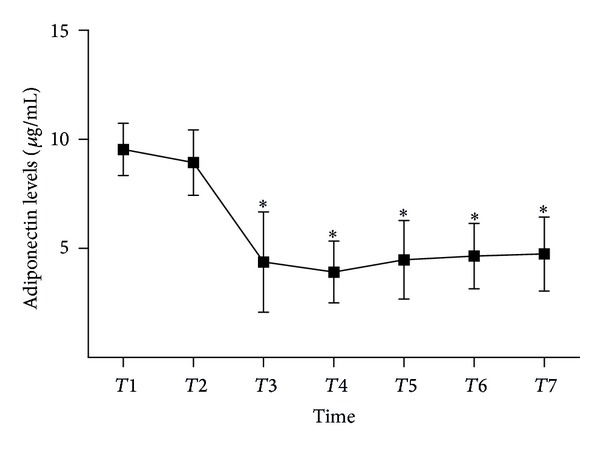
Changes in adiponectin levels in the perioperative period. Reported significances (*P < 0.05 was calculated using pairwise comparisons with the preoperative level within a repeated measurement analysis of variance model for the respective parameter at different time points). The error bars designate the standard deviation (CPB: cardiopulmonary bypass; *T*: time; *T*1: before anesthesia; *T*2: initiation of CPB; *T*3: termination of CPB; *T*4: 6 h after CPB; *T*5: 12 h after CPB; *T*6: 24 h after CPB; *T*7: 48 h after CPB).

 All data were expressed as mean with standard error of the mean. Pearson's correlation coefficient was estimated for associations between adiponectin and metabolic variables at different time points. Repeated measures analysis of variance (ANOVA) models (Figures 1, 2, and 3) were analysed using SPSS version 13.0 (SPSS, Inc., Chicago, IL, USA).

## 3. Results

### 3.1. Characteristics of the Study Group

 Baseline characteristics of the study participants are shown in Table 1. The cardiac surgery included repair of ventricular septal defects in 35 patients, atrial septal defects in 18 patients, and correction of tetralogy of Fallot in 7 patients.

### 3.2. Kinetics of Insulin Resistance

 Blood glucose was monitored on an hourly basis throughout the observation period. All patients required insulin treatment to maintain euglycaemia.  Figure 1(a) shows the stable blood glucose levels throughout the observation period. Serum insulin concentrations increased at the termination of CPB, following the course of exogenously applied insulin, and remained stable thereafter (Figure 1(b)). To create a more specific parameter of insulin resistance that combines serum glucose with serum insulin levels, we calculated an insulin glycaemic index (insulin × glucose/22.5) at each time point (Figure 1(c)). The insulin glycaemic index increased during the first 22 hours of the observation period and remained stable thereafter reflecting the kinetics of exogenously applied insulin.

### 3.3. Kinetics Inflammatory Cytokines

 During the observation period inflammatory cytokines rapidly increased with peak concentrations of TNF-*α* and IL-6 at the 6 h time point (Figures 2(a) and 2(b)). Adiponectin serum levels were repressed throughout the observation period reaching a minimum at the 6 h time point (Figure 3).

### 3.4. Correlations of Adiponectin with Metabolic Variables at Different Time Points

**Figure 4:**
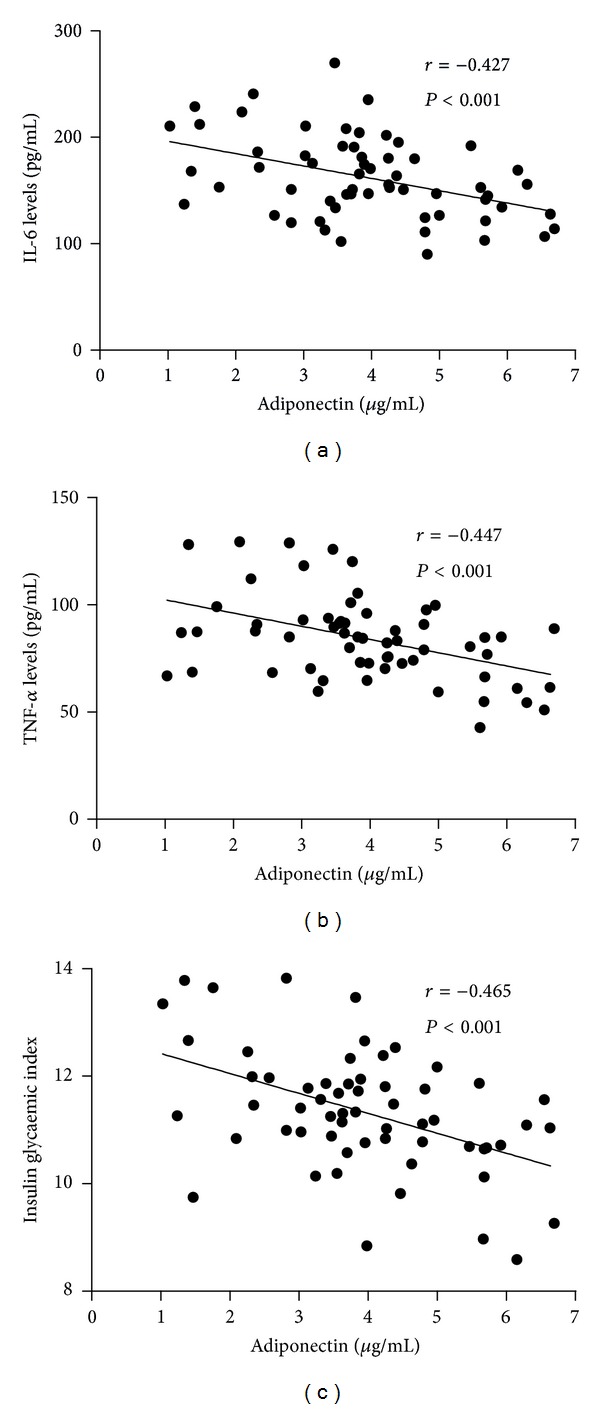
Correlations of adiponectin at *T*4 (6 h after CPB) with IL-6 (a), TNF-*α* (b), and insulin glycaemic index (c). Pearson's correlation coefficient (*r*) and *P* values of the corresponding significance test are both presented.

 There was no association between the adiponectin at *T*3,   *T*5, *T*6, and *T*7 time points and glycemic index, TNF-alpha and IL-6 (Table 2). At *T*4 (6 h after CPB) we found significant inverse correlations of adiponectin with insulin glycaemic index, IL-6, and TNF-*α* (Figure 4). Correlation of adiponectin with the insulin glycaemic index was *r* = −0.465 (*P* < 0.001) was adiponectin with IL-6, *r* = −0.427 (*P* < 0.001), and adiponectin with TNF-*α* was *r* = −0.447 (*P* < 0.001). 

## 4. Discussion

Several studies have reported that adiponectin has a negative correlation with insulin resistance in chronic diseases such as metabolic syndrome and type 2 diabetes [15, 16]. However, the relationship of adiponectin with insulin resistance and inflammatory mediators in infants undergoing cardiac surgery with cardiopulmonary bypass has not been identified so far. The present study demonstrated the correlation of adiponectin with insulin resistance and the kinetic changes of inflammatory cytokines in infants undergoing CPB. CPB provokes a systemic inflammatory response. This inflammatory reaction may contribute to the development of postoperative complications. The marked increases in the amount of exogenous insulin requirement to maintain euglycemia as well as circulating insulin levels during CPB surgery suggest the development of insulin resistance. Our study showed significant increase in TNF-*α* and IL-6 levels after the initiation of CPB and their kinetics at various time points. At the same time, the need of an increased rate of insulin infusion to maintain euglycemia following the operation suggested the development of insulin resistance. Insulin resistance is associated with the inflammatory response, but its molecular basis and physiological significance are not fully understood. Inflammatory mediators such as TNF-*α* and IL-6 either alone or through synergistic effect could lead to the development of insulin resistance by blocking the signal transduction of insulin, impairing insulin sensitivity, and increasing free fatty acids [17, 18]. Insulin resistance would be more intense as inflammatory mediator levels increase.

 Adiponectin has been shown to directly or indirectly affect insulin sensitivity through modulation of insulin signaling and the molecules involved in glucose and lipid metabolism [12]. Adiponectin-deficient mice were shown to be prone to diet-induced obesity and insulin resistance and its reversal by adiponectin treatment [19]. In humans, low adiponectin was more closely associated with insulin resistance than adiposity [20]. In infants undergoing cardiac surgery, IL-6 and TNF-*α* levels were markedly increased while serum adiponectin levels were moderately decreased. This suggests the inverse relationship of circulating adiponectin levels to IL-6 and TNF-*α* and insulin resistance in critically ill patients. The repression of adiponectin serum levels in our model and its association with insulin resistance are in agreement with previous reports [13, 21]. Low adiponectin levels were associated with high inflammatory levels and intense insulin resistance. This indicates the role of adiponectin in regulation of glucose metabolism (insulin resistance) and inflammatory mediators.

## 5. Conclusions

In summary, we have demonstrated the significant inverse association of adiponectin with markers of systemic inflammation and insulin resistance in infants undergoing open cardiac surgery. The better understanding of the association of adiponectin with insulin resistance and systemic inflammation will be of high clinical value as it may have therapeutic implications.

## Figures and Tables

**Table 1:** Baseline characteristics and operative data of infants (*n* = 60).

Characteristics	Data
Male gender (%)	36 (60%)
Age (year)	1.5 ± 0.4
Body weight (kg)	5.9 ± 1.7
Left ventricular ejection fraction (%)	67.4 ± 8.6
Cardiopulmonary bypass time (min)	50.3 ± 7.9
Cross-clamping time (min)	35.4 ± 4.3
Cardiopulmonary bypass flow (L/min/m^2^)	2.8 ± 0.4
Ultrafiltration (mL/kg)	337 ± 32
Insulin (*μ*U/mL)	7.8 ± 1.6
Blood glucose level (mmol/L)	4.6 ± 0.5
Tumor necrosis factor-*α* (pg/mL)	32.7 ± 10.4
Interleukin-6 (pg/mL)	19.9 ± 15.7
Adiponectin (*μ*g/mL)	9.5 ± 1.2

Data are presented as the number (%) of patients or mean values ± SD.

**Table 2:** Correlations of adiponectin with metabolic variables.

	Adiponectin with the insulin glycaemic index	Adiponectin with IL-6	Adiponectin with TNF-*α*
*T*1	−0.415*	−0.397*	−0.419*
*T*2	−0.408*	−0.384*	−0.379*
*T*3	−0.354	−0.347	−0.364
*T*4	−0.465**	−0.427**	−0.447**
*T*5	−0.346	−0.352	−0.357
*T*6	−0.358	−0.371	−0.374
*T*7	−0.361	−0.375	−0.342

Pearson's correlation coefficient (*r*) and *P* values of the corresponding significance test are both presented. (*T*: time; *T*1: before anesthesia; *T*2: initiation of CPB; *T*3: termination of CPB; *T*4: 6 h after CPB; *T*5: 12 h after CPB; *T*6: 24 h after CPB; *T*7: 48 h after CPB. *P < 0.05 and **P < 0.001.)
